# Vitreous hyper-reflective dots and the macular thickness after cataract surgery

**DOI:** 10.1371/journal.pone.0300148

**Published:** 2024-04-09

**Authors:** Jurica Predović, Damir Bosnar, Leon Marković, Ana Ćurić, Josipa Bračić, Thomas Georgi, Wolfgang List, Wilfried Glatz, Domagoj Ivastinovic

**Affiliations:** 1 Department of Ophthalmology, Reference Center of the Ministry of Health of the Republic of Croatia for Pediatric Ophthalmology and Strabismus, University Hospital “Sveti Duh”, Zagreb, Croatia; 2 Faculty of Medicine, Josip Juraj Strossmayer University of Osijek, Osijek, Croatia; 3 Faculty of Dental Medicine and Health Osijek, Josip Juraj Strossmayer University of Osijek, Osijek, Croatia; 4 Department of Ophthalmology, Medical University of Graz, Graz, Austria; Nicolaus Copernicus University, POLAND

## Abstract

**Purpose:**

To assess the association between vitreous hyper-reflective dots (VHD) and the macular thickness changes following uneventful phacoemulsification.

**Methods:**

In this prospective cohort study optical coherence tomography (OCT) examinations were performed preoperatively and 1 week, 1 month and 3 months postoperatively in patients undergoing cataract surgery. OCT images were analyzed for retinal central subfield thickness (CST) and preretinal VHDs. Surgeries were recorded for the assessment of lens fragments in the space of Berger.

**Results:**

111 eyes of 97 patient were enrolled of whom 69 (62.2%) were female. VHDs were seen in 25 eyes (22.5%) at week 1; in 21 eyes (18.9%) at month 1 and in 3 eyes (2.7%) at month 3. In all eyes with VHDs retro-capsular lens fragments were visible immediately after phacoemulsification. The number of VHDs significantly decreased over the postoperative period. There was a moderate correlation between the number of VHDs and CST at 1 month (r = 0.426, p<0.001). In eyes with VHD the CST averaged 238.8±17.6 μm (214–266) at 1 week; 276.1±63.5 μm (231–481) at 1 month and 285.1±122.3 μm (227–785) at 3 months. In eyes with no detectable VHDs CST averaged 235.9±23.3 μm (192–311) at 1 week; 240.1±21.6 μm (200–288) at 1 month and 242.2±21.3 μm (205–289) at 3 months. Although the differences among the assessment points were relatively low, there was a significant difference in general (p<0.001, Friedman test).

**Conclusion:**

In conclusion, VHDs seem to cause macular thickening throughout the postoperative course. The origin of VHDs is still unknown; however, they presumably represent lens fragments that provoke subclinical inflammation.

## Introduction

Cystoid macular edema (CME) can be detected in up to 13.9% of eyes after uneventful phacoemulsification using optical coherence tomography (OCT) [1–7]. Its occurrence is based on the postoperative release of inflammatory mediators causing blood-retina barrier breakdown and the up-regulation of pro-inflammatory genes and proteins in the retina [8–10]. Recently, vitreous hyper-reflective dots (VHDs) were identified as a highly significant risk factor for pseudophakic CME [11, 12]. VHDs presumably represent lens fragments that are formed during phacoemulsification and accelerated through the zonula retro-capsular into the space of Berger [13, 14]. Since decapsulated lens fragments provoke inflammation, VHDs presumably contribute to postoperative inflammation and thus macular thickening including CME [11, 12, 15, 16]. So far, the relationship between VHDs and CME has been studied only retrospectively [11, 12]. The aim of this study is to prospectively assess the association between VHDs and the macular thickness changes following uneventful phacoemulsification.

## Methods

This prospective study obtained ethics committee approval by the Ethics Committee of the Medical University of Graz and was registered in clinicaltrials.gov. The study was conducted in accordance with the principles and regulations of the Declaration of Helsinki. Patients undergoing cataract surgery were recruited and included after receiving their written informed consent. Patients were recruited between July 2018 and June 2019, data collection was terminated by September 2019. OCTs of the posterior segment were performed preoperatively and at 1 week, 1 month and 3 months after the surgery using Spectral domain OCT (Spectralis version 6.0.9 software, Heidelberg Engineering, Heidelberg, Germany). For the purpose of the study we used volume scanning with 25 sections covering a field of 20°x20° in the macular region. The device used a bandwidth of 297 nm and a wavelength of 815 nm. A built-in eye tracking software (TruTrack) ensured the exact position of the recorded scans. Sections were received using the high-speed mode with a resolution of 7 μm axially x 14μm laterally and a distance of 240 μm between sections.

**Fig 1:**
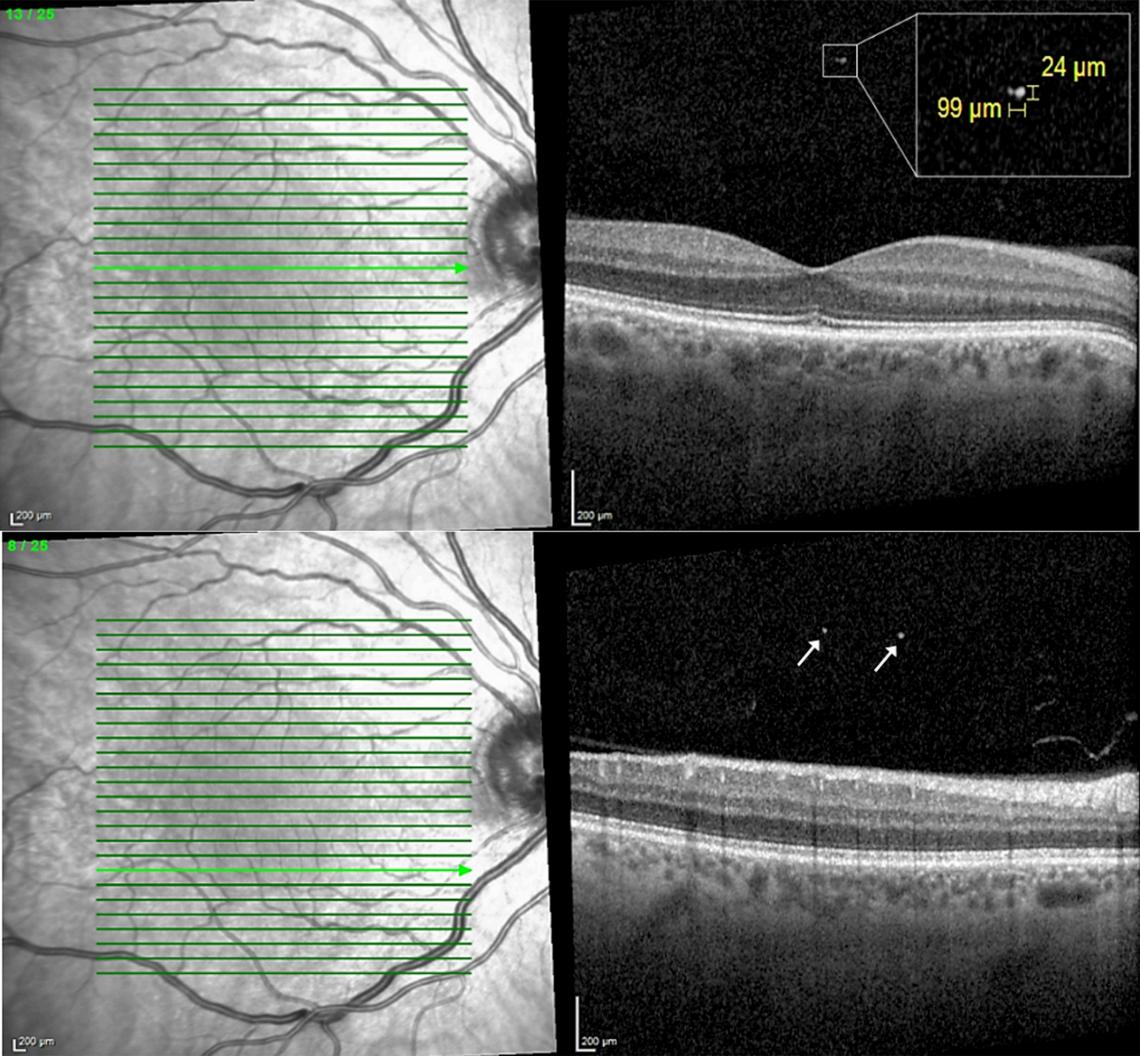
OCT at 1 month after phacoemulsification. (Upper) The rectangle displays a magnified vitreous hyperreflective dot (VHD) with bars measuring its vertical and horizontal diameter. The bar measuring the horizontal diameter appears shorter since the OCT scan is displayed with full vertical resolution (1:1 pixel) instead of the vertical scale adjusted to the horizontal scale (1:1 μm). (Lower) Extrafoveal OCT of the same patient with two VHDs (arrows).

The OCT image included central subfield thickness (CST) in μm and VHDs, defined as clearly visible hyper-reflective dots of variable size of >20 μm in diameter (Fig 1) [11, 12]. Total number of VHDs was determined by summing the VHDs of each section. The diameters were manually assessed using the measurement bars provided by the OCT software (Fig 1). CME was defined as macular thickness >300 μm and the presence of intraretinal hypo-reflective cysts within the ETDRS circle [17]. Exclusion criteria were intraoperative complications including capsule rupture with or without anterior vitrectomy, iris bites; previous interventions including vitrectomy, glaucoma surgery; exudative or dry age-related macular degeneration; presence of diabetes; history or presence of retinal vein occlusion or uveitis; and epiretinal glioses.

**Fig 2:**
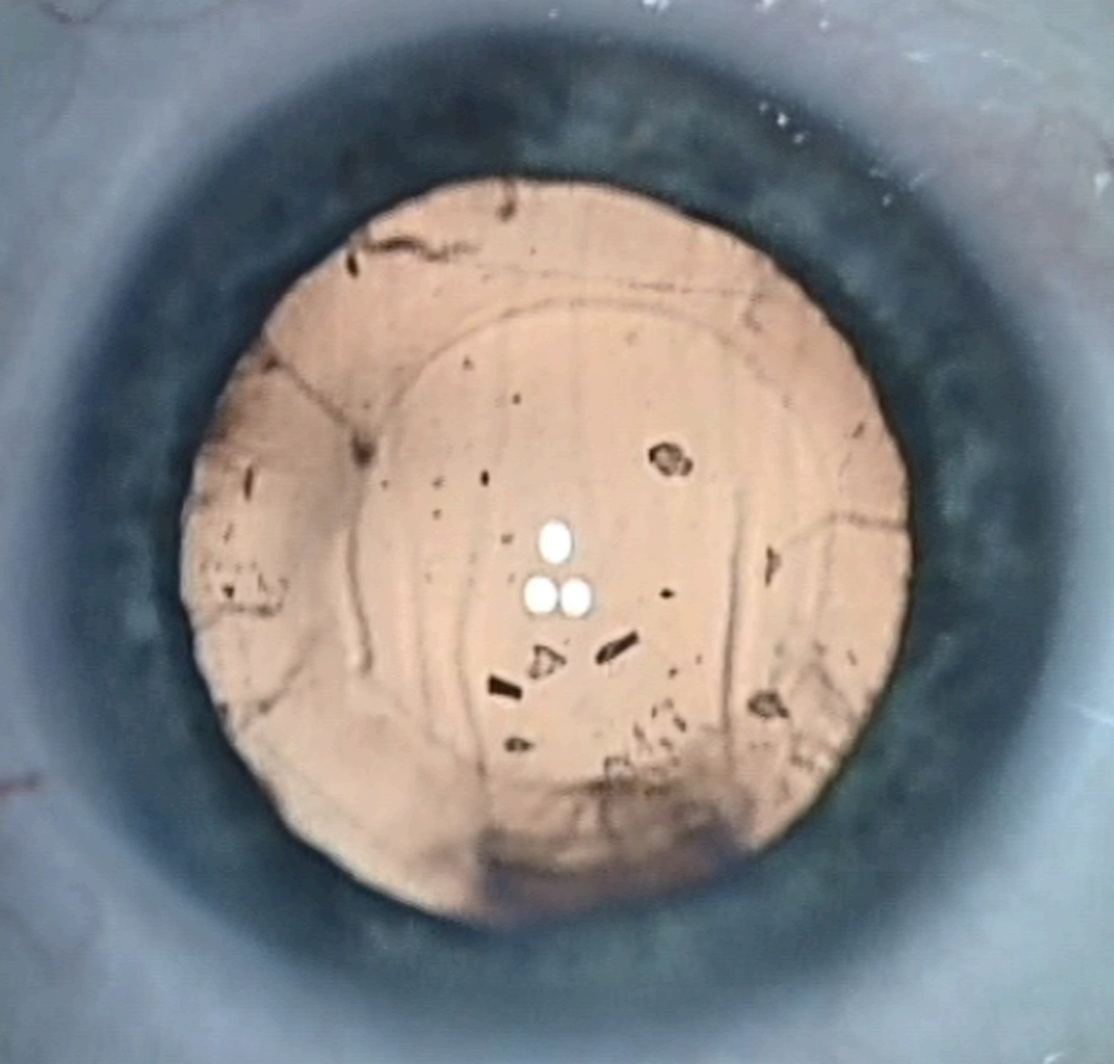
Multiple lens fragments embedded in the retro-capsular space immediately after phacoemulsification.

Prior to surgery, all patients underwent in addition to OCT detailed preoperative ophthalmic examinations including best-corrected visual acuity (BCVA); biomicroscopy with indirect ophthalmoscopy; applanation tonometry; and biometry including keratometry values, axial length, and the anterior chamber depth (IOL-Master 700, Carl Zeiss Meditec, Jena, Germany). Axial length was measured from the corneal vertex to the retinal pigment epithelium by partial coherence interferometry. In case of dense cataract, axial length values were obtained by ultrasound (Axis II PR, Quantel Medical, Clermont-Ferrand, France), measuring the distance between the corneal vertex and the inner limiting membrane of the retina. All patients underwent cataract surgery using the manual anterior continuous curvilinear capsulorhexis phacoemulsification technique for the removal of the crystalline lens and subsequent implantation of a monofocal hydrophobic acrylic intraocular lens in the capsular bag. The surgeries were recorded for the assessment of lens fragments in the retro-capsular region following phacoemulsification (Fig 2). No intraoperative OCT scans were available.

At the end of the surgery, 0.1 ml cefuroxime was injected into the anterior chamber. Postoperatively, all patients received dexamethasone 0.1% drops (Maxidex®, Alcon Laboratories Inc., Geneva, Switzerland) q.i.d. for 7 days followed by b.i.d. for 7 days.

The descriptive data are presented as mean ± standard deviation (range). Normal distribution was assessed with Kolmogorov-Smirnov test. Since the data was not normally distributed, the differences in the continuous data were calculated with the Friedman test (>2 paired samples) or Wilcoxon test (2 paired samples). Intra- and inter-observer differences regarding the number of VHDs were assessed with intraclass correlation coefficients and 95% confidence intervals (CIs). Correlations between various parameters were determined by using Spearman correlation analysis. Jonkheere´s trend test was used to evaluate trends. The statistics were two-tailed. The threshold for significance was defined as p<0.05.

## Results

Overall, 111 eyes of 97 patients could be enrolled in the analysis. Forty-two patients (37.8%) were male, and 69 patients (62.2%) were female. Their age averaged 71.9±9.2 (44–83) years. The mean keratometric value (MKV), the anterior chamber depth (ACD) and the axial length (AL) were 43.6±1.6 diopters (39.7–46.9); 3.1±0.5 mm (2.0–4.8) and 23.1±1.3 mm (20.8–27.2), respectively. The CST averaged 233.8±18.6 μm (197–276) preoperatively; 236.6±22.1 μm (192–311) at 1 week; 248.2±38.4 μm (200–481) at 1 month and 251.8±62.8 μm (205–785) at 3 months. The difference among the assessments in general was significant (p<0.001, Friedman test). In addition, the differences in CST between the preoperative and values at 1 month and 3 months were significant (both p<0.001, Wilcoxon test). No statistical differences were detectable in CST between preoperative and 1 week (p = 0.504, Wilcoxon test) and between 1 month and 3 months (p = 0.264, Wilcoxon test). CME occurred in three eyes (2.6%) at month 1. In one of these eyes, CME persisted throughout the observation period. In 1 eye (0.9%) CME was detected at 3 months.

VHDs were detectable in 25 eyes (22.5%) at week 1; in 21 eyes (18.9%) at month 1 and in 3 eyes (2.7%) at month 3 (Fig 1). This trend towards a lower proportion of eyes with VHDs throughout the duration of the study was significant (p<0.001, Jonkheere´s trend test). The number of VHDs averaged 5.5±3.8 (1–16) at week 1; 4.8±2.7 (1–9) at month 1 and 5.5±3.5 (3–8) at month 3. There was a moderate correlation between the number of VHDs and CST at 1 month (r = 0.426, p<0.001). At other assessment points, these correlations were very weak and statistically not significant (at 1 week r = 0.107, p = 0.299; at 3 months r = 0.182, p = 0.055). In all eyes with VHDs retro-capsular lens fragments were visible immediately after phacoemulsification (Fig 2).

In eyes that showed VHDs at 1 week (n = 25) there was a significant trend towards CST increase (p = 0.018, Jonkheere´s trend test). In detail, in these eyes CST averaged 238.8±17.6 μm (214–266) at 1 week; 276.1±63.5 μm (231–481) at 1 month and 285.1±122.3 μm (227–785) at 3 months. In eyes with no detectable VHDs at 1 week (n = 86), CST averaged 235.9±23.3 μm (192–311) at 1 week; 240.1±21.6 μm (200–288) at 1 month and 242.2±21.3 μm (205–289) at 3 months. Although the differences among the assessment points were relatively low, there was a significant difference in general (p<0.001, Friedman test). In addition, there was also a statistically significant trend towards CST increase (p = 0.026, Jonkheere´s trend test).

## Discussion

Our study shows that after phacoemulsification VHDs occur in up to 22% of eyes with a peak at 1 week and a decreasing proportion over time. In addition, especially those eyes with VHDs at 1 week after the surgery developed a more significant CST increase including CME throughout the observation period compared to those with no VHDs. Also, the number of detected VHDs at 1 month after the surgery seemed to play a role in this context. Accordingly, there was a moderate but highly significant correlation between the number of VHDs and CST at 1 month. Although a correlation does not automatically imply a causal relationship, we assume that our finding indicates a relation between the number of VHDs and the macular thickness to a certain degree. This assumption is also based on a previous finding of a significant correlation between the number of VHDs and CST in eyes with CME [12]. Conversely, there was also a significant trend towards CST increase in eyes with no detectable VHDs at 1 week. However, this trend was weaker compared to those with VHDs although the number of eyes with no VHDs was substantially higher (78.3% vs. 21.7%). This finding indicates that macular thickening is not only related to VHDs. The occurrence of VHDs following cataract surgery was already reported [11, 12]. Accordingly, VHDs were highly predictive for pseudophakic CME with a statistically highly significant odds ratio of 2.4 [11, 12]. Since both studies had inherent limitations due to their retrospective design, we performed this prospective study. Despite the complexity of the development of pseudophakic CME, our study indicates that VHDs seem to play a role in the context of postoperative macular thickness changes.

The exact nature of VHDs can only be assumed since biopsies of VHDs for histopathological evaluations in otherwise healthy eyes would be ethically questionable. Hence, VHDs are assumed to originate from lens fragments produced by phacoemulsification [12]. To confirm this assumption we recorded the surgeries to assess retro-capsular lens fragments after phacoemulsification (Fig 2). Accordingly, all eyes that showed VHDs postoperatively showed retro-capsular lens fragments intraoperatively (Fig 2). Probably, these lens fragments migrate towards the retina postoperatively and become visible with OCT as VHDs. Since decapsulated lens fragments provoke inflammation, VHDs presumably promote an inflammatory response and thus macular thickening [11–15]. The appearance of retro-capsular lens fragments after phacoemulsification was already reported in 17% eyes [15]. However, in this study no association with the macular thickness was evaluated [15]. According to our study, VHDs presumably resolve over months since the proportion of eyes with visible VHDs peaked at 1 week with a decreasing proportion over the following months. Particularly in the period between 1 month and 3 months after the surgery this proportion significantly dropped from 18.3% to 2.7%.

The implication of our study is that eyes with retro-capsular lens fragments observed intraoperatively might benefit from parabulbar/subconjunctival cortisone injections at the end of the cataract surgery to suppress the development of CME as already shown in the ESCRS PREMED Study Report 2 [18]. Certainly, pseudophakic CME is a benign and self-limiting complication in the majority of affected eyes. However, up to 27% of eyes do not reach visual *restitutio ad integrum* despite resolution of pseudophakic CME secondary to subtle alterations of the outer photoreceptor morphology [19]. Hence, applying cortisone parabulbarly or subconjunctivally in eyes with increased risk of macular swelling would be an individualized and proactive approach to decrease the likelihood of CME and preserve an intact retina.

We are aware that VHDs are not the main reason for CME. For example, an older age and renal insufficiency are significant risk factor for CME [11, 12, 20]. Also other factors like pseudoexfoliation and zonular weakness might have contributed to lens fragment dispersion into the vitreous and consequently to the inflammatory response resulting in macular thickening. However, in our previous study, we could not find an association between pseudoexfoliation and CST [12]. Therefore we did not further focus on this issue. Assessments of other potential risk factors would require a much larger study scale.

This study has some limitations. Due to the limited number of eyes, we could not confirm VHDs as a significant risk factor for pseudophakic CME in particular. Admittedly, this was the initial first goal of our study. However, the low CME rate in our prospective analysis counteracted our goal. Nevertheless, our study clearly indicates that VHDs detected at 1 week after the surgery have an impact on the extent of macular thickening over the following months. Another limitation is the assessment of VHDs itself. In detail, one can only assess VHDs with OCT in the preretinal vitreous. Thus, VHDs beyond the detection range of OCT and those between the sections of the OCT image remained undetected. This technical limitation is a known and accepted bias, however [11, 12]. Hyperreflective dots in the vitreous in OCT were discussed in a plurality of previous publications (e.g. diabetic retinopathy, uveitis, floaters, conglomerations of inflammatory cells, epithelial cells from the ciliary body, vitreous collagen fibrils, …). In this study, only VHDs with > 20 μm diameter were considered. For this size lens fragments are the most likely origin for VHDs [12].

In summary, our results suggest that VHDs represent retro-capsular lens fragments and probably contribute to the inflammatory response of the eye to the cataract surgery and consequently impact the extent of postoperative macular thickening. Consequently, parabulbar or subconjunctival cortisone injections at the end of the cataract surgery where retro-capsular lens fragments are visible might be suitable to reduce the risk of pseudophakic CME.

## Supporting information

S1 Checklist *Wie PLOS ONE* clinical studies checklist. (PDF)

S2 Checklist STROBE statement—checklist of items that should be included in reports of observational studies. (PDF)

S3 Checklist TREND statement checklist. (PDF)

S1 File (PDF)

S2 File (PDF)

## References

[1] Ray S, D’Amico DJ. Pseudophakic cystoid macular edema. Semin Ophthalmol 2002. Sep-Dec;17(3–4):167–180. [PubMed] [Google Scholar] 12759847

[2] Yonekawa Y, Kim IK. Pseudophakic cystoid macular edema. Curr Opin Ophthalmol 2012. January;23(1):26–32. doi: 10.1097/ICU.0b013e32834cd5f8 [PubMed] [Google Scholar] 22134362

[3] Henderson BA, Kim JY, Ament CS, Ferrufino-Ponce ZK, Grabowska A, Cremers SL. Clinical pseudophakic cystoid macular edema. Risk factors for development and duration after treatment. J Cataract Refract Surg 2007. September;33(9):1550–1558. doi: 10.1016/j.jcrs.2007.05.013 [PubMed] [Google Scholar] 17720069

[4] Loewenstein A, Zur D. Postsurgical cystoid macular edema. Dev Ophthalmol 2010;47:148–159. doi: 10.1159/000320078 [PubMed] [Google Scholar] 20703048

[5] Cagini C, Fiore T, Iaccheri B, Piccinelli F, Ricci MA, Fruttini D. Macular thickness measured by optical coherence tomography in a healthy population before and after uncomplicated cataract phacoemulsification surgery. Curr Eye Res 2009. December;34(12):1036–1041. doi: 10.3109/02713680903288937 [PubMed] [Google Scholar] 19958122

[6] Biro Z, Balla Z, Kovacs B. Change of foveal and perifoveal thickness measured by OCT after phacoemulsification and IOL implantation. Eye (Lond) 2008. January;22(1):8–12. [PubMed] [Google Scholar] doi: 10.1038/sj.eye.6702460 16751754

[7] Ivastinovic D, Schwab C, Mossbock G, Weger M, Possl SL, Petrovski G, et al. The configuration of the vitreomacular interface determines the pattern of pseudophakic cystoid macular oedema. Acta Ophthalmol 2017. June;95(4):e347–e348. doi: 10.1111/aos.13104 [PubMed] [Google Scholar] 27226394

[8] Miyake K, Ibaraki N. Prostaglandins and cystoid macular edema. Surv Ophthalmol 2002. August;47 Suppl 1:S203–18. [PubMed] [Google Scholar] doi: 10.1016/s0039-6257(02)00294-1 12204717

[9] Ursell PG, Spalton DJ, Whitcup SM, Nussenblatt RB. Cystoid macular edema after phacoemulsification: relationship to blood-aqueous barrier damage and visual acuity. J Cataract Refract Surg 1999. November;25(11):1492–1497. [PubMed] [Google Scholar] doi: 10.1016/s0886-3350(99)00196-0 10569164

[10] Xu H, Chen M, Forrester JV, Lois N. Cataract surgery induces retinal pro-inflammatory gene expression and protein secretion. Invest Ophthalmol Vis Sci 2011. January 5;52(1):249–255. doi: 10.1167/iovs.10-6001 [PubMed] [Google Scholar] 20720227

[11] Oh JH, Chuck RS, Do JR, Park CY. Vitreous hyper-reflective dots in optical coherence tomography and cystoid macular edema after uneventful phacoemulsification surgery. PLoS One 2014. April 15;9(4):e95066 doi: 10.1371/journal.pone.0095066 [PMC free article] [PubMed] [Google Scholar] 24736274 PMC3988138

[12] Glatz W, Steinwender G, Tarmann L, Malle EM, Schörkhuber M, Wackernagel W, et al. Vitreous hyper-reflective dots in pseudophakic cystoid macular edema assessed with optical coherence tomography. PLoS One 2017;12:e0189194. doi: 10.1371/journal.pone.0189194 eCollection 2017. 29244855 PMC5731694

[13] Anisimova NS, Arbisser LB, Shilova NF, Melnik MA, Belodedova AV, Knyazer B, et al. Anterior vitreous detachment: risk factor for intraoperative complications during phacoemulsification. J Cataract Refract Surg 2020. Jan;46(1):55–62. doi: 10.1016/j.jcrs.2019.08.005 32050233

[14] Vael A, Van Os L, Melis K, Tassignon MJ. Evaluation of the vitreolenticular interface with intraoperative OCT. J Cataract Refract Surg 2022. Jul 1;48(7):826–830. doi: 10.1097/j.jcrs.0000000000000866 34775398

[15] Ang A, Menezo i Rallo V, Shepstone L, Burton RL. Retrocapsular lens fragments after uneventful phacoemulsification cataract surgery. J Cataract Refract Surg 2004. April;30(4):849–853. doi: 10.1016/j.jcrs.2003.08.026 [PubMed] [Google Scholar] 15093649

[16] Kim JE, Flynn HW Jr, Rubsamen PE, Murray TG, Davis JL, Smiddy WE. Endophthalmitis in patients with retained lens fragments after phacoemulsification. Ophthalmology 1996. April;103(4):575–578. doi: 10.1016/s0161-6420(96)30651-9 8618754

[17] Kim SJ, Bressler NM. Optical coherence tomography and cataract surgery. Curr Opin Ophthalmol 2009. January;20(1):46–51. [PubMed] [Google Scholar] doi: 10.1097/icu.0b013e3283199162 19093330

[18] Wielders LHP, Schouten JSAG, Winkens B, van den Biggelaar FJHM, Veldhuizen CA, Murta JCN, et al. Randomized controlled European multicenter trial on the prevention of cystoid macular edema after cataract surgery in diabetics: ESCRS PREMED Study Report 2. J Cataract Refract Surg 2018;44:836–847. doi: 10.1016/j.jcrs.2018.05.015 30055692

[19] Hunter A.A., Modjtahedi S.P., Long K., Zawadzki R., Chin E.K., Caspar J.J., et al. Improving visual outcomes by preserving outer retina morphology in eyes with resolved pseudophakic cystoid macular edema. J. Cataract Refract. Surg. 2014;40:626–631. doi: 10.1016/j.jcrs.2013.09.018 24529660

[20] Scheers D, Rens J, Van Os L, Ní Dhubhghaill S, De Groot V, Kiekens S, et al. Safety of the bag-in-the-lens implantation regarding the development of clinically significant pseudophakic cystoid macular edema: A retrospective case series study. PLoS One 2023. Jan 6;18(1):e0278861. doi: 10.1371/journal.pone.0278861 eCollection 2023. 36607976 PMC9821458

