# Supplementary material for: Vitreous hyper-reflective dots and the macular thickness after cataract surgery
Source: PLoS One. 2024 Apr 9;19(4):e0300148. doi: 10.1371/journal.pone.0300148 (PMC11003696; doi:10.1371/journal.pone.0300148)
Supplement: S2 Checklist — (PDF) [file pone.0300148.s002.pdf]

STROBE Statement—checklist of items that should be included in reports of observational studies

	Item No.	Recommendation	Page No.	Relevant text from manuscript
**Title and abstract**	1	(*a*) Indicate the study's design with a commonly used term in the title or the abstract	2	In this prospective cohort study optical coherence tomography (OCT) examinations were performed preoperatively and 1 week, 1 month and 3 months postoperatively in patients undergoing cataract surgery.
		(*b*) Provide in the abstract an informative and balanced summary of what was done and what was found	2	Purpose: To assess the association between vitreous hyper-reflextive dots (VHD) and the macular thickness changes following uneventful phacoemulsification. Methods: In this prospective cohort study optical coherence tomography (OCT) examinations were performed preoperatively and 1 week, 1 month and 3 months postoperatively in patients undergoing cataract surgery. OCT images were analyzed for retinal central subfield thickness (CST) and preretinal VHDs. Surgeries were recorded for the assessment of lens fragments in the anterior vitreous.

Results: 111 eyes of 97 patient were enrolled of whom 69 (62.2%) were female. VHDs were seen in 25 eyes (22.5%) at week 1; in 21 eyes (18.9%) at month 1 and in 3 eyes (2.7%) at month 3. In all eyes with VHDs retro-capsular lens fragments were visible immediately after phacoemulsification. The number of VHDs significantly decreased over the postoperative period. There was a moderate correlation between the number of VHDs and CST at 1 month ($r=0.426$, $p<0.001$). In eyes with VHD the CST averaged 238.8±17.6 µm (214-266) at 1 week; 276.1±63.5 µm (231-481) at 1 month and 285.1±122.3 µm (227-785) at 3 months. In eyes with no detectable VHDs CST averaged 235.9±23.3 µm (192-311) at 1 week; 240.1±21.6 µm (200-288) at 1 month and 242.2±21.3 µm (205-289) at 3 months. Although the differences among the assessment points were relatively low, there was a significant difference in general ($p<0.001$, Friedman test).

**Introduction**				
Background/rationale	2	Explain the scientific background and rationale for the investigation being reported	4	Cystoid macular edema (CME) can be detected in up to 13.9% of eyes after uneventful phacoemulsification using optical coherence tomography (OCT). Its occurrence is based on the postoperative release of inflammatory mediators causing blood-retina barrier breakdown and the up-regulation of pro-inflammatory genes and proteins in the retina. Recently, vitreous hyper-reflective dots (VHDs) were identified as a highly significant risk factor for pseudophakic CME. VHDs presumably represent lens fragments that are formed during phacoemulsification and accelerated through the zonula into the vitreous cavity. Since decapsulated lens fragments provoke inflammation, VHDs presumably contribute to postoperative inflammation and thus macular thickening including CME.
Objectives	3	State specific objectives, including any prespecified hypotheses	4	The aim of this study is to prospectively assess the association between VHDs and

				the macular thickness changes following uneventful phacoemulsification.
**Methods**				
Study design	4	Present key elements of study design early in the paper	4	This prospective study was approved by the local ethic committees (30-273 ex 17/18) and registered in clinicaltrials.gov (NCT05811182) and adhered to the tenets of the Declaration of Helsinki. Patients undergoing cataract surgery were recruited and included after receiving their informed consent.
Setting	5	Describe the setting, locations, and relevant dates, including periods of recruitment, exposure, follow-up, and data collection	4-6	This prospective study was approved by the local ethic committees (30-273 ex 17/18) and registered in clinicaltrials.gov (NCT05811182) and adhered to the tenets of the Declaration of Helsinki. Patients undergoing cataract surgery were recruited and included after receiving their informed consent. Patients were recruited between July 2018 and June 2019, data collection was terminated by September 2019. OCTs were performed preoperatively and at

				1 week, 1 month and 3 months after the surgery using Spectral domain OCT (Spectralis version 6.0.9 software, Heidelberg Engineering, Heidelberg, Germany).<br>Prior to surgery, all patients underwent in addition to OCT detailed preoperative ophthalmic examinations including best-corrected visual acuity (BCVA); biomicroscopy with indirect ophthalmoscopy; applanation tonometry; and biometry including keratometry values, axial length, and the anterior chamber depth (IOL-Master 700, Carl Zeiss Meditec, Jena, Germany).<br>The surgeries were recorded for the assessment of lens fragments in the anterior vitreous.
Participants	6	(*a*) *Cohort study*—Give the eligibility criteria, and the sources and methods of selection of participants. Describe methods of follow-up<br>*Case-control study*—Give the eligibility criteria, and the sources and methods of case ascertainment and control selection. Give the rationale for the choice of cases and controls<br>*Cross-sectional study*—Give the eligibility criteria, and the sources and methods of selection of participants	4,5	Patients undergoing cataract surgery were recruited and included after receiving their informed consent. Exclusion criteria were intraoperative complications including capsule rupture with or without anterior vitrectomy, iris bites; previous interventions including

				vitrectomy, glaucoma surgery; exudative or dry age-related macular degeneration; presence of diabetes; history or presence of retinal vein occlusion or uveitis; and epiretinal gliosis. Follow-up was at 1 week, 1 month and 3 months after the surgery.
		(*b*) *Cohort study*—For matched studies, give matching criteria and number of exposed and unexposed<br>*Case-control study*—For matched studies, give matching criteria and the number of controls per case	N/A	N/A
Variables	7	Clearly define all outcomes, exposures, predictors, potential confounders, and effect modifiers. Give diagnostic criteria, if applicable	4-6	Central subfield thickness (CST) in µm, VHDs defined as clearly visible hyper-reflective dots of variable size of >20 µm in diameter, CME was defined as macular thickness >300 µm and the presence of intraretinal hypo-reflective cysts within the ETDRS circle
Data sources/ measurement	8*	For each variable of interest, give sources of data and details of methods of assessment (measurement). Describe comparability of assessment methods if there is more than one group	4-5	OCT (Spectralis version 6.0.9 software, Heidelberg Engineering, Heidelberg, Germany). For the purpose of the study we used volume scanning with 25 sections covering a field of 20°x20° in the macular region. The device used a bandwidth of 297 nm and

				a wavelength of 815 nm. A built-in eye tracking software (TruTrack) ensured the exact position of the recorded scans. Sections were received using the high-speed mode with a resolution of 7 µm axially x 14µm laterally and a distance of 240 µm between sections. The OCT image included central subfield thickness (CST) in µm and VHDs, defined as clearly visible hyper-reflective dots of variable size of >20 µm in diameter.
Bias	9	Describe any efforts to address potential sources of bias	N/A	N/A
Study size	10	Explain how the study size was arrived at		All patients undergoing cataract surgery and giving written informed consent were included

Continued on next page

Quantitative variables	11	Explain how quantitative variables were handled in the analyses. If applicable, describe which groupings were chosen and why	5	The OCT image included central subfield thickness (CST) in µm and VHDs, defined as clearly visible hyper-reflective dots of variable size of >20 µm in diameter (Fig 1). Total number of VHDs was determined by summing the VHDs of each section. The diameters were manually assessed using the measurement bars provided by the OCT software. CME was defined as macular thickness >300 µm and the presence of intraretinal hypo-reflective cysts within the ETDRS circle.
Statistical methods	12	(*a*) Describe all statistical methods, including those used to control for confounding	6	The descriptive data are presented as mean ± standard deviation (range). Normal distribution was assessed with Kolmogorov-Smirnov test. Since the data was not normally distributed, the differences in the continuous data were calculated with the Friedman test (>2 paired samples) or Wilcoxon test (2 paired samples). Intra- and inter-observer differences regarding the number of VHDs were assessed with intraclass correlation coefficients and 95% confidence intervals (CIs). Correlations between various parameters were determined by

				using Spearman correlation analysis. Jonkheere´s trend test was used to evaluate trends. The statistics were two-tailed. The threshold for significance was defined as p<0.05.
		(*b*) Describe any methods used to examine subgroups and interactions	N/A	N/A
		(*c*) Explain how missing data were addressed		No missing data
		(*d*) *Cohort study*—If applicable, explain how loss to follow-up was addressed<br>*Case-control study*—If applicable, explain how matching of cases and controls was addressed<br>*Cross-sectional study*—If applicable, describe analytical methods taking account of sampling strategy	N/A	N/A
		(*e*) Describe any sensitivity analyses	N/A	N/A
**Results**				
Participants	13*	(a) Report numbers of individuals at each stage of study—eg numbers potentially eligible, examined for eligibility, confirmed eligible, included in the study, completing follow-up, and analysed	6	Overall, 111 eyes of 97 patients could be enrolled in the analysis. Forty-two patients (37.8%) were male and 69 patients (62.2%) were female. Their age averaged 71.9±9.2 (44-83) years. No loss of follow-up.
		(b) Give reasons for non-participation at each stage	N/A	N/A
		(c) Consider use of a flow diagram	N/A	N/A
Descriptive data	14*	(a) Give characteristics of study participants (eg demographic, clinical, social) and information on exposures and potential confounders	6,7	Overall, 111 eyes of 97 patients could be enrolled in the analysis. Forty-two patients (37.8%) were male and 69 patients (62.2%) were female. Their age averaged 71.9±9.2 (44-83) years. The mean keratometric value (MKV), the anterior chamber depth (ACD) and

				the axial length (AL) were 43.6±1.6 diopters (39.7-46.9); 3.1±0.5 mm (2.0-4.8) and 23.1±1.3 mm (20.8-27.2), respectively. The CST averaged 233.8±18.6 µm (197-276) preoperatively; 236.6±22.1 µm (192-311) at 1 week; 248.2±38.4 µm (200-481) at 1 month and 251.8±62.8 µm (205-785) at 3 months. VHDs were detectable in 25 eyes (22.5%) at week 1; in 21 eyes (18.9%) at month 1 and in 3 eyes (2.7%) at month 3. The number of VHDs averaged 5.5±3.8 (1-16) at week 1; 4.8±2.7 (1-9) at month 1 and 5.5±3.5 (3-8) at month 3.
		(b) Indicate number of participants with missing data for each variable of interest	N/A	N/A
		(c) *Cohort study*—Summarise follow-up time (eg, average and total amount)	4,7	Follow-ups: 1 week, 1 month and 3 months postoperatively. Patients were recruited between July 2018 and June 2019, data collection was terminated by September 2019.
Outcome data	15*	*Cohort study*—Report numbers of outcome events or summary measures over time	6,7	CST, VHD, CME
		Case-control study—Report numbers in each exposure category, or summary measures of exposure		
		Cross-sectional study—Report numbers of outcome events or summary measures		
Main results	16	(*a*) Give unadjusted estimates and, if applicable, confounder-adjusted estimates and their precision (eg, 95% confidence interval). Make clear which confounders were adjusted for and why they were included	6,7,8	The CST averaged 233.8±18.6 µm (197-276) preoperatively; 236.6±22.1 µm (192-311) at 1 week; 248.2±38.4 µm (200-481) at 1 month and 251.8±62.8 µm (205-

785) at 3 months. The difference among the assessments in general was significant ($p<0.001$, Friedman test). In addition, the differences in CST between the preoperative and values at 1 month and 3 months were significant (both $p<0.001$, Wilcoxon test). No statistical differences were detectable in CST between preoperative and 1 week ($p=0.504$, Wilcoxon test) and between 1 month and 3 months ($p=0.264$, Wilcoxon test).
VHDs were detectable in 25 eyes (22.5%) at week 1; in 21 eyes (18.9%) at month 1 and in 3 eyes (2.7%) at month 3 (Fig. 1). This trend towards a lower proportion of eyes with VHDs throughout the duration of the study was significant ($p<0.001$, Jonkheere´s trend test). The number of VHDs averaged 5.5±3.8 (1-16) at week 1; 4.8±2.7 (1-9) at month 1 and 5.5±3.5 (3-8) at month 3. There was a moderate correlation between the number of VHDs and CST at 1 month ($r=0.426$, $p<0.001$). At other assessment points, these correlations were very weak and statistically not significant (at 1

			week r=0.107, p=0.299; at 3 months r=0.182, p=0.055). In eyes that showed VHDs at 1 week (n=25) there was a significant trend towards CST increase (p=0.018, Jonkheere´s trend test). In detail, in these eyes CST averaged 238.8±17.6 µm (214-266) at 1 week; 276.1±63.5 µm (231-481) at 1 month and 285.1±122.3 µm (227-785) at 3 months. In eyes with no detectable VHDs at 1 week (n=86), CST averaged 235.9±23.3 µm (192-311) at 1 week; 240.1±21.6 µm (200-288) at 1 month and 242.2±21.3 µm (205-289) at 3 months. Although the differences among the assessment points were relatively low, there was a significant difference in general (p<0.001, Friedman test). In addition, there was also a statistically significant trend towards CST increase (p=0.026, Jonkheere´s trend test). Intra- and inter-observer differences regarding the number of VHDs were assessed with intraclass correlation coefficients and 95% confidence intervals (CIs).
	(*b*) Report category boundaries when continuous variables were categorized	N/A	N/A

	(*c*) If relevant, consider translating estimates of relative risk into absolute risk for a meaningful time period	N/A	N/A

Continued on next page

Other analyses	17	Report other analyses done—eg analyses of subgroups and interactions, and sensitivity analyses	N/A	N/A
**Discussion**				
Key results	18	Summarise key results with reference to study objectives	8,9	Our study shows that after phacoemulsification VHDs occur in up to 22% of eyes with a peak at 1 week and a decreasing proportion over time. In addition, especially those eyes with VHDs at 1 week after the surgery developed a more significant CST increase including CME throughout the observation period compared to those with no VHDs. Also, the number of detected VHDs at 1 month after the surgery seemed to play a role in this context. Accordingly, there was a moderate but highly significant correlation between the number of VHDs and CST at 1 month. There was also a significant trend towards CST increase in eyes with no detectable VHDs at 1 week. However, this trend was weaker compared to those with VHDs although the number of eyes with no VHDs was substantially higher (78.3% vs. 21.7%).
Limitations	19	Discuss limitations of the study, taking into account sources of potential bias or imprecision. Discuss both direction and magnitude of any potential bias	10	This study has some limitations. Due to the limited number of eyes, we could not confirm VHDs as a significant risk factor for

				pseudophakic CME in particular. Admittedly, this was the initial first goal of our study. However, the low CME rate in our prospective analysis counteracted our goal. Nevertheless, our study clearly indicates that VHDs detected at 1 week after the surgery have an impact on the extent of macular thickening over the following months. Another limitation is the assessment of VHDs itself. In detail, one can only assess VHDs with OCT in the preretinal vitreous. Thus, VHDs beyond the detection range of OCT and those between the sections of the OCT image remained undetected. This technical limitation is a known and accepted bias, however.
Interpretation	20	Give a cautious overall interpretation of results considering objectives, limitations, multiplicity of analyses, results from similar studies, and other relevant evidence	11	In summary, our results suggest that VHDs represent retro-capsular lens fragments and probably contribute to the inflammatory response of the eye to the cataract surgery and consequently impact the extent of postoperative macular thickening. Consequently, parabulbar or subconjunctival cortisone injections at the end of the cataract surgery where retro-capsular lens fragments are visible might be suitable to

				reduce the risk of pseudophakic CME.
Generalisability	21	Discuss the generalisability (external validity) of the study results	8	A previous study with retrospective design could show similar results. Also, the occurrence of VHD postoperatively after cataract surgery was previously well described. (Oj JH et al., Glatz W et al.). Measurement of CME, VHD and CST is very well replicable with OCT.
**Other information**				
Funding	22	Give the source of funding and the role of the funders for the present study and, if applicable, for the original study on which the present article is based	N/A	N/A

*Give information separately for cases and controls in case-control studies and, if applicable, for exposed and unexposed groups in cohort and cross-sectional studies.

Note: An Explanation and Elaboration article discusses each checklist item and gives methodological background and published examples of transparent reporting. The STROBE checklist is best used in conjunction with this article (freely available on the Web sites of PLoS Medicine at http://www.plosmedicine.org/, Annals of Internal Medicine at http://www.annals.org/, and Epidemiology at http://www.epidem.com/). Information on the STROBE Initiative is available at www.strobe-statement.org.
