# Supplementary material for: Vitreous hyper-reflective dots and the macular thickness after cataract surgery
Source: PLoS One. 2024 Apr 9;19(4):e0300148. doi: 10.1371/journal.pone.0300148 (PMC11003696; doi:10.1371/journal.pone.0300148)
Supplement: S1 File — (PDF) [file pone.0300148.s004.pdf]

Protokoll:
Prospektive Analyse der Korrelation zwischen hyperreflektiven Dots im Glaskörperraum und der Entwicklung eines pseudophaken zystoiden Makulaödems

Wissenschaftlicher Hintergrund:
In der modernen Kataraktchirurgie wird die Katarakt mittels Ultraschallenergie zerkleinert (Phakoemulsifikation) und abgesaugt, bevor eine artifizielle Linse in das Auge implantiert wird. Hierbei wird die hintere Kapsel der Linse belassen und dient als Aufhängung für die zu implantierende Kunstlinse. Idealerweise dichtet die hintere Kapsel den vorderen Augenabschnitt vom hinteren Augenabschnitt (dem Glaskörperraum) ab. [1] Kommt es zu Einrissen der hinteren Kapsel, kann es zu einer Versprengung von zerkleinertem Linsenmaterial in den Glaskörperraum kommen.
Jong-Hyun et al. haben gezeigt, dass kleine hyperreflektive Punkte in der optischen Kohärenztomographie (OCT), nach Kataraktoperationen auftreten. [2] Bei diesen hyperreflektiven Strukturen dürfte es sich in erster Linie um im Glaskörper befindliches Linsenmaterial handeln. Aufgrund des dadurch initiierten Entzündungsreizes, könnten diese Linsenreste ein wesentlicher Risikofaktor für die Entwicklung einer postoperativen Netzhautschwellung (zystoides Makulaödem - CMÖ) sein. Zu den weiteren, beschriebenen Risikofaktoren für die Entwicklung eines CMÖ zählen Iris Traumata, Austritt von Glaskörper, diabetische Retinopathien, retinale Venenverschlüsse, Pucker Membranen und Uveitis, um einige wenige zu nennen. [2] Diese postoperative zystoide Makulaschwellung wird auch als Irvine-Gass Syndrom beschrieben und tritt im Anschluss an 1% bis 30% der Katarakt Operationen auf. [3] Eine Untersuchung von Glatz et al. hat gezeigt, dass eine positive Korrelation zwischen dem Auftreten von hyperreflektiven Dots im Glaskörperraum und der zentralen Netzhautdicke besteht. [4]
Ziel dieser Arbeit ist es, durch planmäßige OCT Aufnahmen das Auftreten eines postoperativen CMÖ (Irvine-Gass Syndrom) vorzeitig durch hyperreflektive Dots zu erkennen, da diese einem CMÖ vorausgehen können. Hierzu werden eine Woche postoperativ OCT Aufnahmen angefertigt, da das Irvine-Gass-Syndrom üblicherweise erst ab der zweiten postoperativen Woche zu erwarten ist. Anschließend wird in der vierten postoperativen Woche eine OCT Aufnahme angefertigt, da man zu dieser Zeit mit einer vollen Ausprägung des Erkrankungsbilds rechnen kann. Durch diese Aufnahmen lässt sich zeigen, ob aufgrund von hyperreflektiven Dots in der ersten postoperativen Woche das Auftreten eines Irvine-Gass-Syndroms zuverlässig vorausgesagt werden kann und man hier bereits eventuelle therapeutische Maßnahmen setzen sollte. Eine adäquate Therapie würde die intravitreale Applikation von Kortikosteroiden umfassen. [5] Da es sich um eine nicht invasive, kontaktfreie Untersuchung handelt, steht dem recht geringen Risiko ein eventuell hoher Nutzen gegenüber.

Studienplanung - Zielgrößenbeschreibung
Geplant ist eine prospektive Studie. Rund 200 Augen sollen in das Projekt aufgenommen werden. Hierzu werden OCT-Aufnahmen in der ersten und vierten postoperativen Woche nach komplikationsloser Kataraktoperation durchgeführt. Diese werden herangezogen, um die Korrelation

zwischen dem Auftreten, der Anzahl und der Größe von hyperreflektiven Dots im Glaskörperraum und der zentralen Netzhautdicke zu untersuchen. Zusätzlich wird bei der präoperativen Untersuchung eine studienunabhängige OCT Aufnahme durchgeführt, die in die Auswertung miteinbezogen wird. Als Hauptzielgröße wird die Korrelation zwischen den hyperreflektiven Dots im Glaskörperraum vor der zentralen Netzhaut und der zentralen Netzhautdicke formuliert. Dadurch soll in der Zukunft möglicherweise eine bessere Voraussagbarkeit der Wahrscheinlichkeit einer postoperativen Netzhautschwellung gewährleistet sein. Als Nebenzielgrößen werden okuläre Co-Morbiditäten, Bulbuslänge, sowie die Partikelgröße analysiert und ausgewertet.

Auswertung
Die Auswertung unserer Daten erfolgt mit Hilfe der deskriptiven Statistik.

Dauer der Studie
Die Gesamtdauer der Studie soll in etwa 12 Monate betragen

Veröffentlichung der Daten
Die Veröffentlichung der Ergebnisse in einem Peer-reviewed Journal ist vorgesehen.

Beteiligte
Dr.med.univ. Wilfried Glatz, Universitätsklinik für Augenheilkunde
OA Dr.med.univ. Domagoj Ivastinovic, Universitätsklinik für Augenheilkunde
Dr. med.univ. Thomas Georgi, Universitätsklinik für Augenheilkunde
Wolfgang List, Universitätsklinik für Augenheilkunde

Literatur

1. Allen, D., Cataract. BMJ Clinical Evidence, 2011. 02(708).
2. Oh, J.H., et al., Vitreous hyper-reflective dots in optical coherence tomography and cystoid macular edema after uneventful phacoemulsification surgery. PLoS One, 2014. 9(4): p. e95066.
3. Grzybowski, A., et al., Pseudophakic cystoid macular edema: update 2016. Clin Interv Aging, 2016. 11: p. 1221-1229.
4. Glatz, W., et al., Vitreous hyper-reflective dots in pseudophakic cystoid macular edema assessed with optical coherence tomography. PLoS One, 2017. 12(12): p. e0189194.
5. Bonfiglio, V., et al., Widening use of dexamethasone implant for the treatment of macular edema. Drug Des Devel Ther, 2017. 11: p. 2359-2372.
