# Supplementary material for: Vitreous hyper-reflective dots and the macular thickness after cataract surgery
Source: PLoS One. 2024 Apr 9;19(4):e0300148. doi: 10.1371/journal.pone.0300148 (PMC11003696; doi:10.1371/journal.pone.0300148)
Supplement: S2 File — (PDF) [file pone.0300148.s005.pdf]

Study Protocol: Vitreous hyper-reflective dots and the macular thickness after cataract surgery

Scientific Background

In modern cataract surgery, the crystalline lens is dissected using ultrasound energy (phacoemulsification) and aspirated before an artificial lens is implanted into the capsular bag. The capsular bag is left in place and serves as a suspension for the artificial lens to be implanted. Ideally, the posterior capsule seals the anterior segment of the eye from the posterior segment (the vitreous cavity). [1] If tears occur in the posterior capsule, migration of lens fragments into the vitreous cavity may occur.
Jong-Hyun et al. have shown that small hyperreflective dots appear in optical coherence tomography (OCT), after cataract surgery. [2] These hyperreflective structures are likely to be crystalline lens fragments dislocated into the vitreous cavity. Due to the inflammatory stimuli these lens remnants could be a significant risk factor for the development of postoperative retinal thickening (pseudophakic cystoid macular edema - CME). Further assumed risk factors for the development of CMO include iris trauma, vitreous leakage, diabetic retinopathy, retinal vein occlusion, epiretinal membranes, and uveitis, only to name a few. [2] This postoperative cystoid macular thickening is also described as Irvine-Gass syndrome and occurs following 1% to 30% of cataract surgeries. [3] A study by Glatz et al. showed that there is a positive correlation between the occurrence of hyperreflective dots in the vitreous cavity and central retinal thickness. [4]
The aim of this work is to predict the occurrence of postoperative CME (Irvine-Gass syndrome) by screening for hyperreflective dots by scheduled OCTs. For this purpose, OCT images are obtained one week postoperatively since Irvine-Gass syndrome is usually not expected until the second postoperative week. Subsequently, OCT images are obtained four weeks postoperatively, as full expression of CME can be expected at this time. These images show whether the occurrence of Irvine-Gass syndrome can be reliably predicted on the basis of hyperreflective dots in the first postoperative week to enable an early medical therapy. Adequate therapy would include intravitreal application of corticosteroids. [5] Since this is a noninvasive, noncontact examination, the fairly low risk is offset by a possibly high benefit.

Study Design and Outcome Parameters

The study design is a prospective cohort study. 200 eyes will be included. OCT examinations will be performed in the first postoperative week and four weeks postoperatively after uneventful cataract surgery. These will be used to study the correlation between the occurrence, number and size of hyperreflective dots in the vitreous cavity and central retinal thickness. In addition, a study-independent OCT scan will be performed during the preoperative examination and will be included in the analysis. The correlation between hyperreflective dots in the vitreous cavity and central retinal thickness will be formulated as the main outcome measure. This should potentially provide better predictability of the likelihood for postoperative retinal thickening in the future. As a secondary outcome measures, ocular co-morbidities, bulbus length, and hyperreflective dot size will be analyzed and evaluated.

Literature

1. Allen, D., Cataract. BMJ Clinical Evidence, 2011. 02(708).
2. Oh, J.H., et al., Vitreous hyper-reflective dots in optical coherence tomography and cystoid macular edema after uneventful phacoemulsification surgery. PLoS One, 2014. 9(4): p. e95066.
3. Grzybowski, A., et al., Pseudophakic cystoid macular edema: update 2016. Clin Interv Aging,
2016. 11: p. 1221-1229.
4. Glatz, W., et al., Vitreous hyper-reflective dots in pseudophakic cystoid macular edema assessed with optical coherence tomography. PLoS One, 2017. 12(12): p. e0189194.
5. Bonfiglio, V., et al., Widening use of dexamethasone implant for the treatment of macular edema. Drug Des Devel Ther, 2017. 11: p. 2359-2372.
